# A Pilot Study on the Effect of a Self-Training Program on Asian American Caregivers

**DOI:** 10.1093/geroni/igaa057.049

**Published:** 2020-12-16

**Authors:** Tongtong Li, Yufu Jiang, Iris Chi

**Affiliations:** University of Southern California, Los Angeles, California, United States

## Abstract

This presentation reports an exploratory study evaluating the effect of Asian Care Training (ACT) Manual on Asian American (AA) caregivers’ care capacity and knowledge growth. A total of 65 AA caregivers were recruited from community-based organizations in Los Angeles and passed the screening questionnaire, in which 44 (37 females and 7 males with an average age of 61) completed the evaluation process. Participants were given autonomy to choose topics they desired to learn out of 25 topics in ACT manual and completed one-month learning. The data were collected via structured questionnaires before and after the self-learning and were analyzed with paired t-test on 5 questions about care capacity and 4 about caregiving knowledge. One-month self-learning of ACT manual enhanced participants’ care capacity in 4 out of 5 sectors: confidence in caregiving (t=-2.2, p=0.015); ability to deal with emergency (t=-1.88, p=0.032); to solve concerns of the care recipients (t=-4.54, p<0.001); to communicate more smoothly (t=-2.85, p=0.0028). Improvements were found in 3 out of 4 sectors in knowledge: knowledge of Alzheimer’s disease (t=-4.43, p<0.001); appropriated approach to displacing care recipients (t=-5.03, p<0.001); and information about caregiver support resources (t=-7.25, p<0.001). This self-learning method of ACT manual was feasible for AA caregivers to broaden knowledge and care capacity while adapting with their intensive workload. This study constitutes an important step towards establishing culturally tailored self-training programs for Asian American caregivers for frail older adults via multiple platforms such as online curriculums and mHealth app.

